# *Lepidasthenia loboi* sp. n. from Puerto Madryn, Argentina (Polychaeta, Polynoidae)

**DOI:** 10.3897/zookeys.546.6175

**Published:** 2015-12-16

**Authors:** Sergio I. Salazar-Vallejo, Norma Emilia González, Patricia Salazar-Silva

**Affiliations:** 1 El Colegio de la Frontera Sur, Depto. Sistemática y Ecología Acuática, Chetumal, Quintana Roo, México; 2 Instituto Tecnológico de Bahía de Banderas, Nayarit, México

**Keywords:** Thelepodidae, Terebellidae, *Lepidametria*, symbiosis, giant chaetae, Lepidastheniinae

## Abstract

Among polychaetes, polynoids have the highest number of symbiotic species found living with a wide variety of marine invertebrates, including other polychaetes. *Lepidasthenia* Malmgren, 1867 and *Lepidametria* Webster, 1879 were regarded as synonyms but belong to different subfamilies, although both have species associated with thelepodid or terebellid polychaetes. In this contribution *Lepidasthenia loboi* **sp. n.** is described from several specimens associated with the thelepodid *Thelepus antarcticus* Kinberg, 1867, collected on a rocky shore near Puerto Madryn, Argentina. *Lepidasthenia loboi* **sp. n.** can be confused with *Lepidasthenia esbelta* Amaral & Nonato, 1982 because both live with *Thelepus*, are of similar sizes with similar pigmentation patterns, and have giant neurochaetae. However, in *Lepidasthenia loboi* **sp. n.** all eyes are of the same size, cephalic and parapodial cirri are tapered and mucronate, the second pair of elytra is larger than the third, the ventral cirri arise at the base of parapodia such that they do not reach chaetal lobe tips, and neuraciculae are tapered. On the contrary, in *Lepidasthenia esbelta* the posterior eyes are larger than anterior ones, cephalic and parapodial appendages are swollen subdistally, the second and third pairs of elytra are of the same size, the ventral cirri arise medially such that their tips reach the neurochaetal lobe tips, and the neuraciculae have falcate tips. Some comments about other genera in the Lepidastheniinae, a simplified key to its genera, and a key to *Lepidasthenia* species with giant neurochaetae are also included.

## Introduction

The polychaete family Polynoidae has the highest number of species involved in symbiotic relationships (Okuda 1936, Pettibone 1953, Martín and Britayev 1998). They are associated with other invertebrates such as echinoderms, echiurans (Anker et al. 2005), enteropneusts, sipunculans, hermit crabs (Achari 1974), burrowing shrimps (Sato et al. 2001), mollusks, octocorals, and tube-dwelling polychaetes belonging to the families Capitellidae, Chaetopteridae, Maldanidae and Terebellidae (Gibbs 1969).

There are few detailed studies on the relationships between polynoids, and their thelepodid or terebellid hosts. From a physiological perspective, Morgan (1974) studied the interaction between the thelepodid *Thelepus crispus* Johnson, 1901 and the polynoid *Halosydna brevisetosa* Kinberg, 1856 which can also be free-living. His main results were that 1) each thelepodid hosts a single polynoid; 2) their body size is proportional to each other indicating a long-term relationship; and 3) free-living polynoids of the same species do not react to the thelepodids hosting commensal members of the same species, such that contact is made by random encounter. McDermott (2005) found that specimens of *Lepidametria commensalis* Webster, 1879 were found in 65% of the terebellid *Amphitrite ornata* (Leidy, 1855) tubes he collected (42% also had a pinnotherid crab), but no further details on terebellids were included because pinnotherid crabs were the main area of interest.

Gravier (1905), Potts (1910), and Fauvel (1917) regarded *Lepidasthenia* and *Lepidametria* as synonyms, but they have also been regarded as distinct genera (Salazar-Silva 2006, 2009). Their delineation has been confused and remains unsettled. For example, Day (1973:6) indicated that he had earlier (Day 1962:634) “… gave my reasons for regarding *Lepidametria* as a synonym of *Lepidasthenia*. Dr. Pettibone, who is making an intensive study of the Polynoidae informs me that *Lepidametria* is a valid genus and in deference to her opinion I have not changed the name of *Lepidametria commensalis*.” Barnich and Fiege (2004) regarded *Lepidametria* as a valid genus in a recent revision, including a list of genera, key to genera, and comparative tables for Lepidastheniinae Pettibone, 1989. However, *Lepidametria* was not included in the subfamily because their parapodia differ from those of typical Lepidastheniinae; instead they placed it in the subfamily Lepidonotinae. Some additional comments are necessary for clarifying the current status of both genera and, once better defined, changes will be needed for the corresponding species lists in WoRMS (Fauchald 2014a, b).

There are no world-wide keys to species of *Lepidasthenia* Malmgren, 1867 or *Lepidametria* Webster, 1879. There are some keys available (Chamberlin 1919, Fauvel 1923, Seidler 1923), which are in need of updating, and there are some other later ones for species from Japan (Imajima and Hartman 1964), the temperate Eastern Pacific (Ruff 1995), and tropical America (Salazar-Silva 2009).

Some authors have dealt with the delineation of what we now regard as Lepidastheniinae, either by making direct comments on some morphological attributes or by indirect indications of their relevance by including them in keys. Their ideas, especially when they are convergent, are followed in the following sections, especially those made by Seidler (1923), Averintsev and Ushakov (1977), Uschakov (1982), Hanley and Burke (1991), Salazar-Silva (2006, 2009), and Wehe (2006).

In this contribution, *Lepidasthenia loboi* sp. n. is described from specimens collected on a rocky shore nearby Puerto Madryn, Argentina, associated with the thelepodid *Thelepus antarcticus* Kinberg, 1867. Remarks on other genera in the Lepidastheniinae, together with some others about morphology, a simplified key to lepidastheniin genera, and a key to *Lepidasthenia* species with giant neurochaetae are also included.

## Material and methods

**Field sampling.** Cerro Avanzado is a large coastal mountain nearby Puerto Madryn, Argentina, and the same name applies to the nearby shore. There, a long sandy beach is bordered by rocky outgroups; rocks are mudstones which are easily bored through or broken apart. A hammer was used to crack apart larger rocks into fist-sized portions which were brought to the Centro Nacional Patagónico (CENPAT) facilities where specimens were removed from these fragments. Polychaetes were placed in tap water to relax them, then fixed in a 10% formalin solution. After 24 h they were rinsed with tap water and were preserved in 70% ethanol.

**Specimens.** Polynoids, thelepodids and terebellids were identified at El Colegio de la Frontera Sur (ECOSUR). Plates were arranged by compressing a series of digital photographs with HeliconFocus.

**Type specimens deposition.** Type and non-type specimens were deposited in the following institutions:

ECOSUR Colección de Referencia, El Colegio de la Frontera Sur, Chetumal, México.

LACM Allan Hancock Foundation Polychaete Collection, Natural History Museum of Los Angeles County, Los Angeles, U.S.A.

MACN Museo Argentino de Ciencias Naturales Bernardino Rivadavia, Buenos Aires, Argentina.

MZUSP Museo de Zoologia, Universidad Federal de São Paulo, Brazil.

ZUEC Museo de Zoologia, Universidad Estadual de Campinas, Campinas, Brazil.

**Restrictions for keys.** There is one key to Lepidastheniinae genera available (Barnich and Fiege 2004). The present key, by contrast, is simpler to follow as it does not require complete specimens. However, a restriction was made to include only those species provided with giant neurochaetae, which are also present in *Polynoe elegans* Grube, 1840, the type species for *Lepidasthenia*.

## Results

### Morphological characters

**Body.** The number of chaetigers can be useful but because this number changes as the animals grow, and their bodies are delicate and fragment easily, this cannot be a diagnostic feature. Likewise, pigmentation and its intensity might be size-dependent as well and vary according to how long specimens have been stored in alcohol. However, there are some interesting differences regarding pigmentation patterns involving a series of pigmented segments, their number, and the relative shape of segmental bands, although growth abnormalities or regeneration might slightly alter these patterns.

**Prostomium.** The shape and relative length of antennae are useful diagnostic features; antennae can be tapered or subdistally swollen. The relative size of eyes, being either of about the same size, or one pair markedly larger than the other, together with their position on the prostomial surface (variable in relation to lateral margins) are also useful. The relative length of antennae has not been used but could help to separate similar species, with caution as they can sometimes be lost or undergoing regeneration, such that observations on more than a single specimen are desirable.

**First chaetiger.** Some species have an anterior projection over the prostomium, and its surface and margins can be papillated or smooth. Further, in *Lepidametria* species there are notochaetae in the tentaculophore.

**Parapodia.** Parapodial cirri can be basally swollen, tapered or subdistally swollen, and the relative length and width of cirrophores *versus* cirrostyles are relevant as well. It is useful to note the relative size of dorsal and ventral cirri to each other, and to the tip of neurochaetal lobes. Parapodial surfaces are usually smooth in *Lepidasthenia*, often papillated in *Lepidametria*, and rugose, or markedly folded in *Perolepis*. The presence of parapodial papillae is used to group similar polynoid genera (Pettibone 1977), and we think their spatial arrangement could be useful to separate species as well.

**Elytra.** The body can have elytra along its length, or only through to medial chaetigers (*Branchipolynoe*). For those having elytra along the body, their relative size as an indication of how much of dorsum is covered, or if they abut successive or other elytra along the middorsal line, are diagnostic features. In general, most elytra of *Lepidasthenia* are small, often markedly reduced from chaetigers 2–3, whereas in *Lepidametria* they can be of about the same size along the body, either touching along the middorsal line, or leaving a wide dorsal area uncovered. In the posterior region the elytra and cirri can be alternating, or elytra can be present on every three segments. Elytral pigmentation patterns are also useful since it can be solid or homogeneous, black, grayish or pale, have a pigmented spot near the junction or insertion region, or form a band from the insertion region.

**Chaetae.** Notochaetae are present in many *Lepidametria* species, at least along anterior chaetigers, but never present in *Lepidasthenia* species. Neurochaetae of different shapes and relative width are present in *Lepidasthenia*, although they are usually of decreasing length from the superior to the inferior most. They can be finely dentate with a single series of marginal teeth, or more frequently provided with a series of paired rounded stiff blades, each blade with finely denticulate margins, as originally indicated by Potts (1910:344), by Moore (1903:420) who called them transverse combs, and by Chamberlin (1919:50) who referred to them as lanceolate scales. They have been regarded as spines as well, but when seen from the front, they have a wide distal area, not a narrower one as is usually the case for typical spines, and the margins are not smooth but crenulate or denticulate. Neurochaetal tips can be entire or bidentate, and sometimes there can be one pair of lateral subdistal spines or teeth; the relative size of distal teeth and the orientation of their tips can also be diagnostic. Most chaetae are yellowish and of similar width, but sometimes superior chaetae are markedly thicker and darker, honey or light brown in color; some authors have called them giant chaetae.

## Systematics

### Family Polynoidae Kinberg, 1856

#### Subfamily Lepidonotinae Willey, 1902

##### Lepidametria

Taxon classificationAnimaliaPhyllodocidaPolynoidae

Webster, 1879

Lepidametria (incl. *Nectochaeta* von Marenzeller, 1892, *Harmopsides* Chamberlin, 1919, and *Bouchiria* Wesenberg-Lund, 1949).

###### Type species.

*Lepidametria commensalis* Webster, 1879, by monotypy.

###### Diagnosis

(modif. Webster 1879). Body long with up to 80 segments. Elytrae large, covering body or leaving a narrow dorsal surface uncovered; posterior region with elytra and cirri alternating every other segment. Tentaculophores with chaetae. Notopodia reduced, with fine notochaetae at least along anterior and median segments, rarely absent. Neuropodia projecting with several types of neurochaetae. Ventral surface often papillated.

###### Remarks.

*Lepidasthenia* and *Lepidametria* were regarded as synonyms by Gravier (1905), Potts (1910), Fauvel (1917), and Day (1962). Gravier indicated that the main difference was the presence of notochaetae in the latter but at the same time, he apparently rejected this difference by indicating that “*Lepidasthenia elegans* Grube a précisément une rame dorsale rudimentaire”. [Transl.: *Lepidasthenia elegans* precisely has a rudimentary dorsal branch]. However, the diagnostic feature of missing notochaetae does not denote the presence of a notopodial lobe, but rather a complete absence of notochaetae. Consequently, we think that this is an important difference that can be used to easily separate these two genera. This same approach has been useful for separating other genera, and has been implied by the keys by Fauvel (1923:87), or directly by Fauchald (1977:59).

The pattern of the presence of elytra on posterior segments in *Lepidametria* and *Lepidasthenia* made Uschakov (1982), and Hanley and Burke (1991) regard them as distinct genera; i.e., *Lepidametria* has alternating elytra and cirri, whereas *Lepidasthenia* has elytra on every third segment in medial and posterior regions. Barnich and Fiege (2004) indicated they were following these conclusions and regarded *Lepidametria* as a member of Lepidonotinae, not Lepidastheniinae. Their reason was that in *Lepidametria* (Barnich and Fiege 2004:864): “parapodia differ significantly in their shape from those of the members of the Lepidastheniinae. In the Lepidastheniinae neuropodia are well developed, rather elongate, and distinctly notched dorsally and ventrally, while in *Lepidametria* neuropodia are well developed, but shorter, and not distinctly notched, which is typical for the Lepidonotinae Willey, 1902”.

It is unfortunate that there is no redescription for the type species of *Lepidametria*. The only illustrations available do not show this lepidastheniin notch; however, one figure shows that their neuropodia are not short (Pettibone 1963:21, fig. 4g, k). In the original description Webster (1879:12) indicated (italics added): “Ventral ramus of foot stout, elongate, conical, *widely excavated for the transmission of the setae*, and obliquely truncated from above downward.” This phrase in italics could be taken as equivalent to a notched neuropodium, however. The presence of chaetae in the tentaculophore, another non-lepidastheniin feature, was overlooked by Webster, and by Pettibone (1963:20), who regarded *Lepidametria* as a valid genus. Gardiner (1976, fig. 1n) illustrated the presence of chaetae in the tentaculophores, but his figure was based upon non-topotype specimens. After the syntype material was examined by one of us (PSS), we concluded that in *Lepidametria commensalis* there are chaetae in the tentaculophore and that parapodia differ from those present in Lepidastheniinae. Consequently, *Lepidametria* does not belong in this subfamily but in Lepidonotinae, as previously indicated by Barnich and Fiege (2004).

#### Subfamily Lepidastheniinae

Taxon classificationAnimaliaPhyllodocidaPolynoidae

Pettibone, 1989

##### Type genus.

*Lepidasthenia* Malmgren, 1867, by original designation.

##### Diagnosis

(Modif. Barnich and Fiege 2004). Prostomium with median and lateral antennae; lateral antennae terminal, ceratophores distinct. Tentaculophores without chaetae. Palps visible dorsally. Pharynx with jaws and border papillae. Dorsal tubercles indistinct. Dorsal cirrophores without filamentous organs, sometimes with lateral projections. Notopodia reduced, notochaetae usually missing. Neuropodia distally truncate, elongate, notched dorsally and ventrally, forming subequal anterior and posterior lobes; no supra-acicular processes.

##### Remarks.

Pettibone (1989:301) listed six genera as belonging to Lepidastheniinae: *Alentiana* Hartman, 1942, *Benhamipolynoe* Pettibone, 1970, *Hyperhalosydna* Augener, 1922, *Lepidasthenia*, *Perolepis* Ehlers, 1908, and *Telolepidasthenia* Augener & Pettibone in Pettibone, 1970. The review by Barnich and Fiege (2004) is followed regarding the composition and affinities in Lepidastheniinae.

##### Key to genera of Lepidastheniinae Pettibone, 1989

**Table:** 

1	Body with elytrae continued through posterior segments; sometimes reduced in size in medial and posterior segments	**2**
–	Body with elytra limited to anterior and medial regions	**10**
2	Notochaetae present	**3**
–	Notochaetae absent; parapodial surface usually smooth	**4**
3	Lateral antennae with ceratophores as long as wide; dorsal cirri about three times longer than ventral ones (numerous segments, up to 90 pairs of elytra)	***Lepidastheniella* Monro, 1924**
–	Lateral antennae with ceratophores twice as long as wide; dorsal cirri 6–7 times longer than ventral ones (reduced number of segments, 15 pairs of elytra)	***Parahalosydna* Horst, 1915**
4	Elytra alternate with cirri in medial and posterior regions	**5**
–	Elytra present every third segment in medial and posterior regions	**7**
5	First chaetiger with a middorsal anterior projection over prostomium; neurochaetae unidentate with two subdistal teeth; elytra smooth	***Showapolynoe* Imajima, 1997**
–	First chaetiger without anterior projection; elytra with microtubercles	**6**
6	Neurochaetae mostly bidentate with series of 10–20 lamellae; elytral microtubercles along exposed area	***Hyperhalosydna* Augener, 1922**
–	Neurochaetae only unidentates with series of about 10 tiny lamellae; elytral microtubercles scattered	***Benhamipolynoe* Pettibone, 1970** (partim)
7	Elytrophores elongated, pedunculate; ventral cirri sometimes irregularly swollen	***Perolepis* Ehlers, 1908**
–	Elytrophores short, not transformed into peduncles; ventral cirri tapered or subdistally swollen	**8**
8	Medial segments with large elytra, overlapping successive ones or approaching middorsally	**9**
–	Medial segments with tiny, non-overlapping elytra	***Lepidasthenia* Malmgren, 1867**
9	Eyes small, on prostomial upper surface; ventral parapodial surface papillate; all neurochaetae of a single type, with about 20 series of lamellae	***Telolepidasthenia* Pettibone, 1970**
–	Eyes large, on prostomial margins; ventral parapodial surface smooth; neurochaetae of three types: lamellate, denticulate and smooth	***Alentiana* Hartman, 1942**
10	Notochaetae present; parapodial surface smooth; neurochaetae with rows of large lamellae	***Pseudopolynoe* Day, 1962**
–	Notochaetae absent; parapodial surface rugose; neurochaetae with rows of tiny lamellae	***Benhamipolynoe* Pettibone, 1970** (partim)

#### Lepidasthenia

Taxon classificationAnimaliaPhyllodocidaPolynoidae

Malmgren, 1867

##### Type species.

*Polynoe elegans* Grube, 1840, by monotypy.

##### Diagnosis

(modif. Seidler 1923). Body long with up to 150 segments. Elytra small, not covering each other, leaving dorsal region mostly uncovered; posterior region with one pair of elytra every three segments. Each elytron rounded, margins entire, without tubercles, pale or pigmented. Tentaculophores without chaetae. Notopodia reduced, without notochaetae. Neuropodia projecting with several types of neurochaetae. Ventral surface usually smooth.

##### Remarks.

There was some confusion regarding the presence of notochaetae, but in the original diagnosis for *Lepidasthenia*, Malmgren (1867:15–16) indicated: “Ramus superior pedis perminutus acicula sola praeditus, setis omnino carens.” [Transl. Notopodium with minute acicula, chaetae entirely lacking]. An extended diagnosis was provided by Barnich et al. (2012: 406–407).

It must be emphasized that what can be regarded as the *Lepidasthenia* elytra-cirri pattern in the posterior region is shared by *Perolepis* and *Telolepidasthenia*. However, in *Perolepis* species the integument is usually rugose, and at least the first elytrophores are hypertrophied into distinct peduncles or stems, whereas in *Lepidasthenia* the integument is smooth and all elytrophores are reduced. Furthermore, in *Telolepidasthenia* elytrae are large, covering most of the dorsum, and all neurochaetae are unidentate, whereas in *Lepidasthenia* only the first elytra are large enough to touch each other and the remaining ones are reduced exposing the dorsum, and the dentition of the neurochaetal tips is variable.

#### Lepidasthenia loboi sp. n.

Taxon classificationAnimaliaPhyllodocidaPolynoidae

http://zoobank.org/75B1A4B8-4684-49AD-87EA-922AFC4384F2

Figures 1 , 2

Lepidasthenia esbelta : Barnich et al. 2012: 406–407 (*non* Amaral & Nonato, 1982).

##### Type material.

**Southwestern Atlantic, Argentina.** Cerro Avanzado, 16 km southward from Puerto Madryn (42°49'S, 65°04'W), Golfo Nuevo. Holotype (ECOSUR 176), and 12 paratypes, rocky shore, intertidal, in mudstone, within tubes of *Thelepus antarcticus* Kinberg, 1867, coll. 27 Feb. 2013, J.M. Orensanz, N.E. González & S.I. Salazar-Vallejo [Paratypes: Two (ECOSUR 177), 45–64 mm long, 5–6 mm wide, 83–97 chaetigers; two paratypes (LACM 7040), 30–32 mm long, 4 mm wide, 63–77 chaetigers; two paratypes (MACN), 40–58 mm long, 4.0–4.5 mm wide, 78–90 chaetigers; three paratypes (MZUSP 2857), 12–42 mm long, 2–4 mm wide, 37–83 chaetigers; two paratypes (ZUEC 17781, 17782), 36–39 mm long, 4 mm wide, 79–80 chaetigers.

##### Additional material.

**Southwestern Atlantic, Argentina.** One specimen (ECOSUR), San Antonio Oeste (40°44'S, 64°57'W), Golfo San Matías, 3 m, coll. 10 Oct. 2005, J.M. Orensanz (30 mm long, 4 mm wide, 70 chaetigers). Six anterior fragments (ECOSUR), Cerro Avanzado, 16 km southward from Puerto Madryn (42°49'S, 65°04'W), rocky shore, intertidal, in mudstone, with *Thelepus antarcticus* Kinberg, 1867, coll. 27 Feb. 2013, J.M. Orensanz, N.E. González & S.I. Salazar-Vallejo.

##### Description.

**Figure 1.**
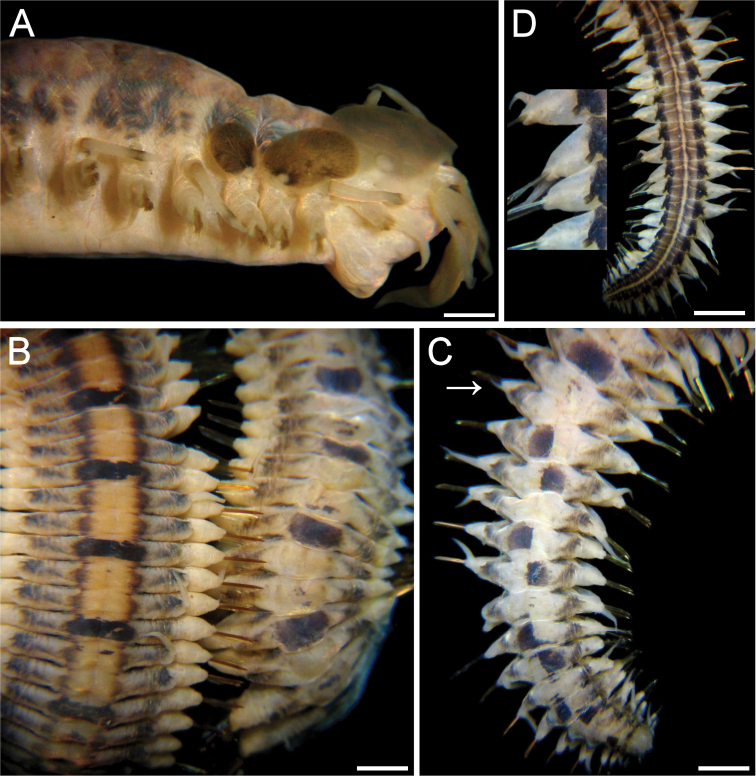
*Lepidasthenia loboi* sp. n., holotype (ECOSUR 176) **A** Anterior end, right lateral view **B** Dorsal surfaces, medial (left) and posterior (right) regions **C** Dorsal surface, posterior end (arrow points to an asymmetrical parapodium) **D** Ventral surface, posterior end (inset: close-up of right parapodia). Bars: 1.1 mm (**A**), 0.5 mm (**B, D**), 1.3 mm (**C**).

Holotype (ECOSUR 176) twisted, almost complete (without anal cirri). Body 64 mm long, 5 mm wide (at chaetiger 1, without chaetae), 99 chaetigers. Antennae, palps and tentacular cirri pale. Dorsal cirri with blackish cirrophore, cirrostyles with subdistal blackish ring, tips pale (Fig. 1A). Dorsum with almost continuous thick, lateral, longitudinal dark-brown bands; bands continuous in chaetigers 1–4, medial areas paler; chaetiger 5 pale, alternating with blackish transverse band (Fig. 1B, C). First transverse band (chaetiger 6) slightly longer than corresponding segment, followed by irregular transverse bands occupying slightly more than half of segment length along 6 chaetigers, middorsal areas with irregular brownish spots, bands then alternating to chaetiger 21, thereafter darker bands every three segments but intermediate segments paler, maculated. First elytra greyish, largest (Fig. 1A); following ones blackish, markedly smaller. Venter smooth; anterior third pale, posterior two-thirds with discontinuous darker, blackish bands along nephridial lobes areas; midventral region slightly less pigmented, ventral chord area paler (Fig. 1D). Far posterior segments with darker pigmentation ventrally. Nephridial papillae projecting, dark.

**Figure 2.**
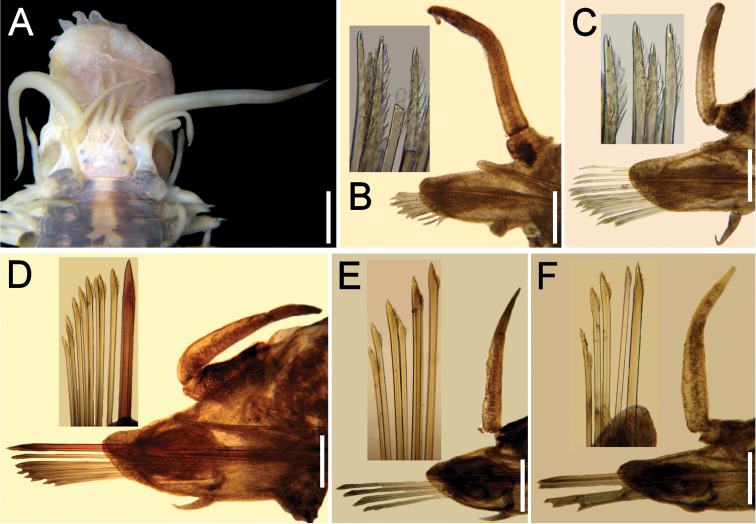
*Lepidasthenia loboi* sp. n. **A** Paratype (ECOSUR 177b), anterior end, dorsal view, first two pairs of elytra removed **B** Paratype (ECOSUR 177a), chaetiger 2, right parapodium, anterior view (inset: neurochaetal tips) **C** Same, chaetiger 9, right parapodium, anterior view (inset: neurochaetal tips) **D** Same, chaetiger 29, right parapodium, anterior view (inset: neurochaetae) **E** Same, chaetiger 59, right parapodium, anterior view, larger chaetae broken (inset: neurochaetae) **F** Same, chaetiger 80, right parapodium, anterior view (inset: neurochaetae). Bars: 0.1 mm (**A**), 40 µm (**B–D**), 45 µm (**E**), 25 µm (**F**).

Prostomium with eyes black, medium-sized (as wide as antennal width), central on prostomium; anterior eyes more separated than posterior ones (Fig. 2A). Medial antenna slightly longer than laterals; ceratophores of similar width, slightly longer than wide; ceratostyles tapered, with long tips. Palps 3–4 times thicker than antennae, right one 2.5 times longer than medial antenna, left one regenerating. Segment 1 with tentacular cirri 1.3 times as long as, and slightly thicker than, antennae, tapered, with long tips mucro.

Elytra on segments 2, 4, 5, alternating with dorsal cirri to chaetiger 26, thereafter on every three segments but last 7 segments more irregular. First pair of elytra largest, covering prostomium and middorsal region, grayish, slightly darker around junction area, laterally with a paler, thin area. Second pair of elytra blackish, oval, less than half as large as first elytra, slightly overlapping anterior elytra, not covering middorsal region, laterally with a paler, thin area. Third pair of elytra blackish, subcircular, less than half as large as second elytra, non-overlapping with previous elytra, not covering middorsal region. Following elytra with same pigmentation, progressively reducing in size, up to chaetiger 20, about twice as large as junction area.

Parapodia sub-biramous throughout body. Notopodia reduced to a projecting, digitate lobe, reducing in size posteriorly. Neuropodia projecting lobes throughout body, neurochaetal lobes truncate or rounded. Dorsal cirri with cirrophores blackish, about as long as wide, cirrostyles tapered, with long tips, longer anteriorly, slightly reducing in length and pigmentation posteriorly, about twice as long as neuropodium. Ventral cirri small, tapered, basal-half blackish, tips mucronate, arising at base of parapodia, about as long as half neuropodial length.

Notopodia without notochaetae. Neurochaetae of different size and shape. Anterior chaetigers with about 15 neurochaetae per bundle, of similar width, smaller ventrally, each with bidentate tips, accessory tooth smaller, directed distally, and 10 or more series of subdistal lamellae (Fig. 2B, C). Medial chaetigers with one giant (thicker, more than twice as wide as other ones), brownish, superior neurochaeta with series of 5–6 tiny spines, tips unidentate, and about 10 thinner bidentate chaetae with series of 5–6 lamellae, becoming thinner and smaller ventrally (Fig. 2D, E). Posterior neuropodia with 1–2 slightly wider, superior chaetae and 4–5 thinner neurochaetae (Fig. 2F). Neuraciculae hyaline, tapered.

Posterior region tapered; pygidium truncate, anus dorsal. Nephridial papillae from chaetiger 9; anterior region with papillae pale, smaller along anterior body half, progressively larger and darker in posterior body half.

Pharynx (observed in some paratypes) with marginal prismatic papillae, upper ones larger, 9 upper and 9 lower. Two pairs of dark brown jaws.

##### Etymology.

This species name honors the late José María Orensanz, in recognition of his many contributions to the study of Southwestern Atlantic and Antarctic polychaetes, of his continued support of our research dreams, and for his participation in the field trip that collected the species. The specific epithet is derived from his nickname, Lobo, and is a noun in apposition.

##### Type locality.

Cerro Avanzado rocky shore, intertidal, Puerto Madryn (42°49'S, 65°04'W), Golfo Nuevo, Argentina.

##### Variation.

Paratypes 12–64 mm long, 2–6 mm wide, 37–99 chaetigers. Smallest specimen with transverse bands restricted to anterior region; larger specimens more heavily pigmented and showing variation in the amount of spots or darkening of paler areas between successive transverse bands. Intensity of pigmentation increased in larger specimens, and in some (including holotype), posterior region had an irregular pattern probably due to imperfect regeneration, which is rather uncommon in other errant polychaetes (Yáñez-Rivera and Méndez 2014).

##### Remarks.

*Lepidasthenia loboi* sp. n. has been confused with *Lepidasthenia esbelta* Amaral & Nonato, 1982, described from southern Brazil because both live with *Thelepus*, have similar size and pigmentation patterns, and giant neurochaetae. However, they differ in several diagnostic features such as the size of eyes, the type of cephalic and parapodial appendages, size of anterior elytra, topology of parapodial cirri, and tips of neuraciculae. In *Lepidasthenia loboi* sp. n. eyes are of the same size, cephalic and parapodial cirri have long tapered tips, second pair of elytra is larger than third, ventral cirri arise basally to neuropodia such that they do not reach the tips of the chaetal lobe, and neuraciculae are tapered. On the contrary, in *Lepidasthenia esbelta* posterior eyes are larger than the anterior ones, cephalic and parapodial appendages are subdistally swollen, the second and third pairs of elytra are of the same size, ventral cirri are medially placed such that their tips reach the tips of the neurochaetal lobe, and the neuroaciculae have falcate tips.

Another species has been recorded from Brazil by Nonato and Luna (1970), and by Amaral and Nonato (1982): *Lepidasthenia virens* (Blanchard in Gay, 1849). These records indicate a lepidastheniin without notochaetae that resembles *Lepidasthenia loboi* because of the type of antennae and tentacular cirri, although palps are shorter than antennae, and by the relative size and position of parapodial cirri, although they illustrated a mature female with hypertrophied dorsal cirrophores. They gave no further detail and the affinities between these two species cannot be clarified. However, two issues deserve comments.

First, *Lepidasthenia virens* was described briefly with material from Calbuco (41°46'S, 73°08'W), Chiloé, Chile. The description and illustration indicates that elytra are large enough to touch each other along the body but while leaving the middorsal surface uncovered (Blanchard 1849:16, Pl. 1, Fig. 2: “… dejando descubierta la porción del medio del dorso, y en cuanto á la longitud del cuerpo apenas si se cubren”). Ehlers (1901:54, Pl. 3, Figs 10–16) described *Lepidasthenia irregularis* with material from the same locality; this species has elytra touching each other, leaving the middorsal surface bare, and notochaetae are present in anterior parapodia. If *Lepidasthenia virens* and *Lepidasthenia irregularis* are synonyms then they both belong in *Lepidametria* by having notochaetae and large elytra overlapping or touching successive ones.

Second, Hartman (1939: 46) noticed this synonymy but because she studied material from a more tropical region, her illustrations do not match Ehlers’ ones. Her specimens have no notochaetae, and neurochaetae are very abundant (ca. 20 per bundle *vs* about 10 per bundle). Consequently, the Eastern tropical Pacific material belongs to another, probably undescribed species, and they differ from true *Lepidasthenia virens* (? = *Lepidasthenia irregularis*).

##### Ecological notes.

*Thelepus antarcticus* Kinberg, 1867 builds its tubes with a mucoid protein forming a semi-transparent matrix with attached fragments of shells or other calcareous fragments. Tubes run inside rock crevices or fractures and are difficult to track individually because they break when the rock is fragmented. There were 34 *Thelepus antarcticus* specimens plus six belonging to two other terebellid species, making it the most frequent thelepodid (or terebellid) in the rocky intertidal environment. About half of *Lepidasthenia loboi* specimens remained inside *Thelepus* tubes, whereas the others left the tubes as the rock was broken. In total, there were 19 *Lepidasthenia loboi* specimens taken at Cerro Avanzado, and there were polynoids in only one-third of the *Thelepus* tubes, half the rate of association between *Thelepus crispus* and *Halosydna brevisetosa* found by Morgan (1974). It would be interesting to conduct a more detailed study to find out what are the functional relationships between *Thelepus antarcticus* and *Lepidasthenia loboi* sp. n. Some specimens exhibited regeneration of palps, antennae, or both, indicating there must be some interactions with other invertebrates, possibly other scale-worms. Some of the anterior fragments were dissected for gut contents but none were found.

##### Distribution.

The specimens were found in two localities in two southern Argentina Gulfs: Cerro Avanzado, Puerto Madryn, Golfo Nuevo, and San Antonio Oeste, Golfo San Matías, but might co-occur with *Thelepus antarcticus* throughout its distribution. Kinberg (1867: 345) described *Thelepus antarcticus* from the intertidal in York Bay, Bucket Island, Magellan Strait. Hartman (1966:109) and Rozbaczylo et al. (2006: 83) regarded it as a junior synonym of *Thelepus plagiostoma* (Schmarda, 1861: 41), described from New Zealand. However, this synonymy was not based upon a study of type material so these two species must be regarded as distinct until a future comparison involving type specimens indicates otherwise. The distribution of *Thelepus antarcticus* would correspond to Patagonian shores, along southern Chile and Argentina, in intertidal to shallow water bottoms.

##### Key to species of *Lepidasthenia* Malmgren, 1867 with giant neurochaetae

**Table:** 

1	Anterior eyes larger than posterior ones	**2**
–	Anterior eyes smaller or subequal to posterior ones	**3**
2	Dorsal cirri subdistally swollen; ventral cirri digitate	***Lepidasthenia elegans* (Grube, 1840)**, Mediterranean Sea
–	Dorsal cirri tapered; ventral cirri basally swollen, tapered	***Lepidasthenia ornata* Treadwell, 1937**, Western Mexico*
3	Dorsal cirri subdistally swollen; ventral cirri tapered, surpassing neurochaetal lobe tip; neuroaciculae falcate	***Lepidasthenia esbelta* Amaral & Nonato, 1982**, Brazil
–	Dorsal and ventral cirri tapered, ventral cirri short, not reaching neurochaetal lobe tip; neuraciculae tapered, straight	***Lepidasthenia loboi* sp. n.**, Patagonia

* A junior synonym of *Lepidasthenia virens* (Blanchard in Gay, 1849) *fide* Hartman (1956: 271); they are probably different. *Lepidasthenia virens* was described from Chiloé, southern Chile, whereas *Lepidasthenia ornata* is from western Mexico. If *Lepidasthenia virens* is the same as *Lepidasthenia irregularis* Ehlers, 1901, both described from the same locality in Chile, and having large notopodia, the latter provided with notochaetae, then both belong in *Lepidametria*.

## Supplementary Material

XML Treatment for Lepidametria

XML Treatment for Lepidastheniinae

XML Treatment for Lepidasthenia

XML Treatment for Lepidasthenia loboi
